# Curriculum Frameworks and Educational Programs in AI for Medical Students, Residents, and Practicing Physicians: Scoping Review

**DOI:** 10.2196/54793

**Published:** 2024-07-18

**Authors:** Raymond Tolentino, Ashkan Baradaran, Genevieve Gore, Pierre Pluye, Samira Abbasgholizadeh-Rahimi

**Affiliations:** 1 Department of Family Medicine McGill University Montreal, QC Canada; 2 Schulich Library of Physical Sciences, Life Sciences, and Engineering McGill University Montreal, QC Canada; 3 Mila - Quebec AI Institute Montreal, QC Canada; 4 Lady Davis Institute for Medical Research Herzl Family Practice Centre Jewish General Hospital Montreal, QC Canada; 5 Faculty of Dental Medicine and Oral Health Sciences McGill University Montreal, QC Canada

**Keywords:** artificial intelligence, machine learning, curriculum, framework, medical education, review

## Abstract

**Background:**

The successful integration of artificial intelligence (AI) into clinical practice is contingent upon physicians’ comprehension of AI principles and its applications. Therefore, it is essential for medical education curricula to incorporate AI topics and concepts, providing future physicians with the foundational knowledge and skills needed. However, there is a knowledge gap in the current understanding and availability of structured AI curriculum frameworks tailored for medical education, which serve as vital guides for instructing and facilitating the learning process.

**Objective:**

The overall aim of this study is to synthesize knowledge from the literature on curriculum frameworks and current educational programs that focus on the teaching and learning of AI for medical students, residents, and practicing physicians.

**Methods:**

We followed a validated framework and the Joanna Briggs Institute methodological guidance for scoping reviews. An information specialist performed a comprehensive search from 2000 to May 2023 in the following bibliographic databases: MEDLINE (Ovid), Embase (Ovid), CENTRAL (Cochrane Library), CINAHL (EBSCOhost), and Scopus as well as the gray literature. Papers were limited to English and French languages. This review included papers that describe curriculum frameworks for teaching and learning AI in medicine, irrespective of country. All types of papers and study designs were included, except conference abstracts and protocols. Two reviewers independently screened the titles and abstracts, read the full texts, and extracted data using a validated data extraction form. Disagreements were resolved by consensus, and if this was not possible, the opinion of a third reviewer was sought. We adhered to the PRISMA-ScR (Preferred Reporting Items for Systematic Reviews and Meta-Analyses extension for Scoping Reviews) checklist for reporting the results.

**Results:**

Of the 5104 papers screened, 21 papers relevant to our eligibility criteria were identified. In total, 90% (19/21) of the papers altogether described 30 current or previously offered educational programs, and 10% (2/21) of the papers described elements of a curriculum framework. One framework describes a general approach to integrating AI curricula throughout the medical learning continuum and another describes a core curriculum for AI in ophthalmology. No papers described a theory, pedagogy, or framework that guided the educational programs.

**Conclusions:**

This review synthesizes recent advancements in AI curriculum frameworks and educational programs within the domain of medical education. To build on this foundation, future researchers are encouraged to engage in a multidisciplinary approach to curriculum redesign. In addition, it is encouraged to initiate dialogues on the integration of AI into medical curriculum planning and to investigate the development, deployment, and appraisal of these innovative educational programs.

**International Registered Report Identifier (IRRID):**

RR2-10.11124/JBIES-22-00374

## Introduction

The field of medicine is constantly evolving with new technologies and discoveries [1]. One of the emerging technologies is artificial intelligence (AI), a simulation of human intelligence powered by machines, specifically computer systems that use machine learning and deep learning [2]. AI allows for complex decision-making and the ability for human capabilities such as tasks done by physicians and other health care providers [2]. Through recent advancements, AI has begun to become an innovation to be adopted in the field of medicine [3]. Current fields using this type of technology are radiology [4], pathology [5], dermatology [6], primary care [7], and surgery [8], among other fields of medicine [9]. These AI-related medical innovations can be seen through different ways, including robot-assisted surgical procedures, diagnosis and risk assessments, as well as the development and customization of drugs [3,10]. However, to move forward with the implementation of AI in clinical practice, physicians need to have a better understanding of AI and how to use it in clinical practice [11].

Although medicine has seen major changes over the last decades, medical education is still largely based on traditional curricula [12]. It often lacks fundamental concepts and even basic familiarization with AI and other emerging technologies [13]. A recent survey by Stanford Medicine found that 44% (230/523) of physicians and 23% (48/210) of medical students and residents reported that their education had not been helpful in preparing for new technologies in health care [14]. Currently, there are no accreditation requirements related to AI [15]. The knowledge gap between engineers, clinicians, and scientists continue to grow as health care moves to a more digital environment, which will ill-prepare young physicians who will work with AI-enabled tools and technologies [16,17].

At the moment, AI is beginning to enter the field of medical education through its uses in learning support, assessments of students’ learning, and curriculum review [2]; however, there are several publications urging institutes and clinical educators to begin integrating AI educational concepts into their medical curricula [12,13,15-20]. There have been efforts to include AI education globally within each level of medical training. These efforts are led by national medical associations such as the UK National Health Service [21], the US American Medical Association [22], and Canada’s Royal College of Physicians and Surgeons [23]. They have released documents recommending policies for integrating AI within their respective medical educational institutions [21-23]. This highlights the importance of the work on the intersection of medical education and AI around the world. Surveys of medical trainees have also supported the need to incorporate the teaching of AI in the undergraduate medical curriculum [24]. To our knowledge, there are no medical schools with formal required courses on AI in health care. While still uncommon, the importance of AI medical education has been identified and acted on at some institutions, such as Duke University, which offers a training course called *Machine Learning School for the School of Medicine* [12]. Other institutions have also developed elective courses to teach AI to residents, such as in radiology [25]. As AI is being used in a variety of fields within medicine [9], it is important to have a structured and validated curriculum framework because future medical providers will be exposed to these types of technologies depending on their chosen fields.

A curriculum framework is a document which describes “the educational environment in which syllabuses (or subject-specific outlines of objectives, outcomes, content and appropriate assessment and teaching methodologies) can be developed” [26]. Curriculum frameworks can be described as educational road maps to teaching and learning. For example, a curriculum framework was created for global health concepts in family medicine education [27]. Medical educators work regularly with frameworks to inform the appropriate learning, assessment, and performance of the health care workforce [28]. Frameworks are tools that can inform the delivery of teaching and curricula development as well as inspire innovation in health care education. There are various aspects that can be included in curriculum frameworks and how they may be used for other disciplines. Obadeji [29] clearly describes the common elements of curriculum frameworks for health professional education, which include (1) the need and the purpose of a curriculum or a program, (2) learning objectives and outcomes, (3) course content that will facilitate the accomplishment of the objectives or learning outcomes, (4) organization of the content, and (5) implementation of curriculum—educational strategies and methods of assessment.

Due to the broad nature of this topic and its prospective limited data, a scoping review is the most appropriate method. Previous reviews exploring topics surrounding AI and medical education have focused on the application of AI in medical education [2,30], attitudes of medical students toward AI [31], and gaps of AI learning within medical education [32]. A recent review of AI educational programs and competencies for health care professionals was published [33]; however, due to the increase in attention on this topic, further reviews must be conducted. Furthermore, the previous reviews had some limitations, such as the exclusion of continuing professional education and the lack of investigating learning theories, pedagogies, and frameworks of their identified AI educational programs. Our review will cover these limitations by focusing on the medical education continuum as the developed AI educational programs for medical students, residents, and practicing physicians can help medical educators navigate the learning pathway for current and future physicians. Moreover, no review has focused on examining curriculum frameworks that guide AI concepts within medical education.

Thus, we conducted a scoping review of published literature on AI curricula being used in medical education. Overall, the main aim of this scoping review is to synthesize knowledge from the literature on curriculum frameworks and current educational programs that focus on the teaching and learning of AI for medical students, residents, and practicing physicians. More specifically, we aim to investigate the details of the current educational programs including (1) the framework, pedagogy, or theory used; (2) the delivery of the educational program; (3) the curricular content; and (4) the evaluation of the program, to inform future research on developing or adopting AI curriculum frameworks for use in medical educational institutions.

## Methods

### Protocol and Registration

The protocol for this review was developed in accordance with the Joanna Briggs Institute (JBI) Reviewers Manual for Evidence Synthesis [34] and guided by the methodological framework developed by Arksey and O’Malley [35], supplemented by Levac et al [36]. The PRISMA-ScR (Preferred Reporting Items for Systematic Reviews and Meta-Analyses extension for Scoping Reviews) [37] was used when reporting results, and is reported in Multimedia Appendix 1. The protocol was registered on Open Science Framework Registries and published on JBI Evidence Synthesis [38].

### Eligibility Criteria

#### Participants

To be eligible for inclusion, the participants of the studies had to fall under the population that provided medical education or received medical education, which includes medical students. This includes undergraduate medical education (UME), residents or postgraduate medical education (PGME), and practicing physicians (continuing medical education [CME]) at any health care setting (ie, primary, secondary, and tertiary care).

#### Exposure

Included studies must describe either a curriculum framework or programs for AI education within medicine. The frameworks and programs must focus on learning about AI and how to use AI-specific tools for the medical profession.

#### Outcome

For the purpose of this review, all elements of a curriculum framework described by Obadeji [29], either in part or as a whole, were considered and reported. Included papers may also describe current and developed educational programs for AI training in medicine. These educational programs have already been developed or evaluated, and papers describing recommendations of what to teach or programs not yet developed were not considered. This review focused on any framework, theory, or pedagogy mentioned within the program; the delivery of the educational program (eg, course and workshop); and curricular content (eg, learning topics and learning objectives); if the educational program was evaluated, it was described according to the model of training evaluation developed by Kirkpatrick et al [39].

### Information Sources

All types of studies were included, such as theoretical work, program descriptions, and empirical studies. Commentaries, reviews, perspectives, opinions, as well as position papers and any companion papers associated were also included. All study designs for empirical studies using qualitative, quantitative, or mixed methods studies were eligible for inclusion. These include experimental and quasi-experimental studies (such as randomized controlled trials, quasi-randomized controlled trials, nonrandomized clinical trials, interrupted time series, and controlled before-and-after studies), observational studies (such as cohort, case control, cross-sectional, and case series studies), qualitative studies (such as ethnography, narrative, phenomenological, grounded theory, and case studies), and mixed methods studies. Conference abstracts and protocols were excluded. Conference abstracts often contain preliminary findings that may not be as comprehensive or validated as full-text articles. As they are brief summaries of studies, they may lack the detailed methodology and results needed for a thorough understanding and synthesis in our scoping review. Furthermore, as protocols are plans of how to conduct the research, they do not provide findings or results that are necessary for a scoping review’s goal to map the extent, range, and nature of research activity in a given field. Therefore, considering the provided justifications, we decided to exclude conference abstracts and protocols.

### Search Strategy

The following search strategy has been developed by a specialized librarian. The text words contained in the titles and abstracts of relevant papers and the index terms used to describe the papers were used to develop a full search strategy. The search strategy took an iterative approach, initially using general terms such as “artificial intelligence,” with the later addition of variations and synonyms such as “deep learning” and “machine learning.” In addition, terms for the concepts of medical education and curriculum were added. An initial limited search of MEDLINE (PubMed) was conducted to identify relevant papers on this topic. An information specialist (GG) performed a comprehensive search in the following bibliographic databases: Ovid MEDLINE, Ovid Embase, CENTRAL (Cochrane Library), CINAHL, and Scopus. To identify any unpublished frameworks, web searches of Google, New York Academy of Medicine Grey Literature Report, and medical learning institutional websites were searched. Reference lists of all included research papers and all relevant reviews were back searched, and Google Scholar was used for forward citation tracking to identify further studies.

Papers were restricted to English and French due to the constraints of the research team. Papers were also restricted by date beginning in the year of 2000, as during the 1950s to the late 1990s AI was in its early phase with reduced funding and interest of AI in medicine [40]. The initial search was conducted in November 2021 and later updated in May 2023.

### Selection of Sources of Evidence

Following the search, all identified records were collated and uploaded into a reference management system, EndNote (version 20.3; Clarivate Analytics), where duplicates were removed. Following a pilot test with 2 reviewers (RT and AB) using 10% (510/5104) of the studies, titles and abstracts were then screened using Rayyan, a web-based research platform, by 2 independent reviewers (RT and AB) for assessment against the inclusion criteria for the review. The full text of selected citations was assessed in detail against the inclusion criteria by 2 independent reviewers (RT and AB). Any disagreements that arose between the 2 reviewers were resolved by a third reviewer (SAR).

### Data Extraction

Data were extracted by 2 reviewers (RT and AB) using a data extraction tool on an Excel (Microsoft Corp) sheet developed and validated by the team. The data extraction tool was created and validated using previously validated data extraction tools [32-34] and input from experts in the field. It focuses on key characteristics related to curriculum framework elements and educational program details. Any disagreements that arose between the 2 reviewers were resolved by a third reviewer (SAR). Data on paper characteristics (eg, authors, title, country of origin, type of study, and year of publication), curriculum framework elements, and educational program details were extracted.

### Synthesis of Results

The results of the review are presented as a table of the data extracted from the included literature to highlight the key findings with respect to the aims of this scoping review. Descriptive statistics (eg, frequency) was used when reporting paper characteristics and education program details. For curriculum frameworks described, reviewers presented main elements, including (1) the need and purpose of curriculum, (2) the learning objectives and outcomes, (3) course content that will facilitate the accomplishment of the objectives or learning outcomes, (4) the organization of the content, and (5) implementation of curriculum. For current educational programs described, reviewers independently recorded and presented data on the framework, theory, or pedagogy that may have been used; the delivery of the educational program; and curricular content; and if the educational program was evaluated, it was described according to the model of training evaluation developed by Kirkpatrick et al [39].

**Figure 1:**
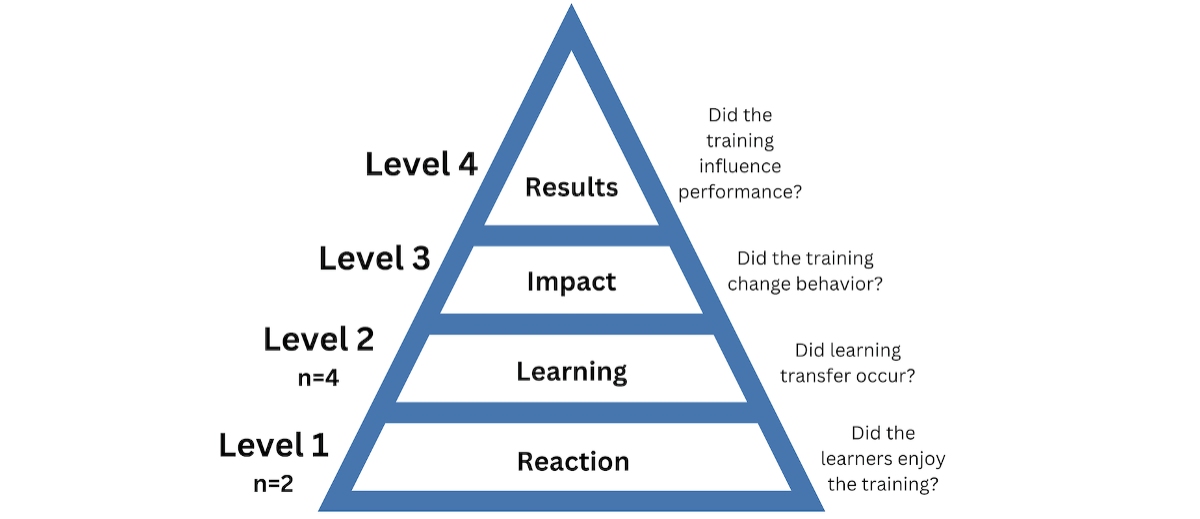
Outcomes (and their meaning) of the 4-level training evaluation developed by Kirkpatrick and Kirkpatrick [39].

The model of training evaluation developed by Kirkpatrick et al [39] was used to categorize educational outcomes evaluations (Figure 1 [39]). Level 1 describes the degree to which learners find the training favorable, engaging, and relevant; level 2 describes the degree to which learners acquire the intended knowledge, skills, confidence, and commitment based on their participation in the training; level 3 describes the degree to which learners apply what they learned during training when they are back to work; and level 4 describes the degree in whether the targeted outcomes resulted from the training program at an organizational level [39]. A narrative summary accompanied [41] the charted results and described what and how AI curriculum content is being delivered to trainees of various medical education stages.

### Quality Appraisal of Included Studies

Due to the nature of this review, the methodological quality or risk of bias of the included papers was not appraised, which is consistent with scoping reviews guidelines [34,37].

## Results

### Search Results

**Figure 2:**
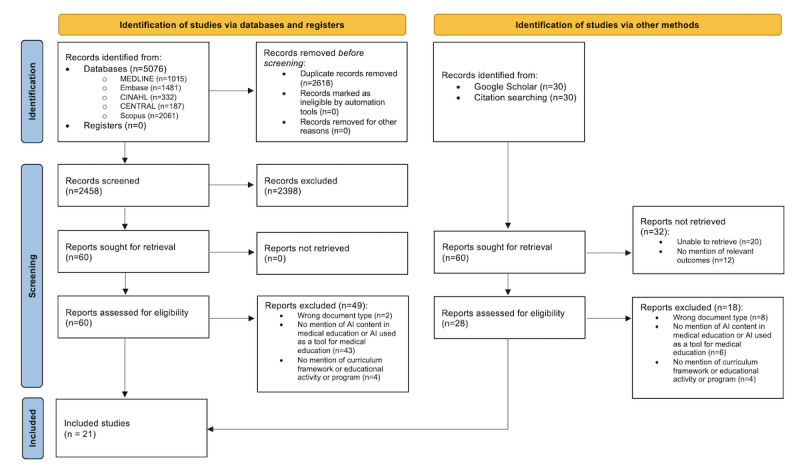
PRISMA (Preferred Reporting Items for Systematic Reviews and Meta-Analyses) flowchart.

From the systematic search, 5076 total papers were identified. These papers were extracted from web-based databases, and the computer software EndNote was used to manage these references. Following removal of duplicates on EndNote, 2458 papers were uploaded to Rayyan software and screened by title and abstract. After abstract and title screening, 60 papers remained for full-text screening. A gray literature search identified 60 papers from Google Scholar and reference lists, from which 28 (47%) papers were retrieved for full-text screening, and 32 (53%) papers were not retrieved or were irrelevant. Following full-text screening of databases and gray literature, 21 papers were included for further analysis [12,25,31-33,42-57]. Refer to the PRISMA (Preferred Reporting Items for Systematic Reviews and Meta-Analyses) diagram (Figure 2) [58].

### Characteristics of the Included Studies

Data was collected from 21 included studies and summarized in Multimedia Appendix 2 [12,25,31-33,42-57]. A total of 12 studies were published in the United States [12,31,32,42-45,48,51,52,54,57]; 6 in Canada [33,46,47,49,50,55]; and 1 each in Germany [25], Korea [53], and Oman [56] (Multimedia Appendix 3). The earliest publication retrieved was from 2016, with 77% (15/21) of the papers [25,31-33,42,43,45,47,49-51,54-57] published in the last 3 years since the pandemic began (Multimedia Appendix 3). From the 21 studies, 6 (29%) were reviews [31-33,45,53,54], 4 (19%) were commentaries [44,47,50,51], 4 (19%) were opinions [12,48,52,56], 3 (14%) were perspectives [43,55,57], 3 (14%) were empirical studies using a cross-sectional survey design [25,42,49], and 1 (5%) was a position paper [46].

In terms of setting, 43% (9/21) of the papers mentioned multiple levels of education ranging from UME, PGME, to CME [12,31-33,46,50,51,54,56], while 24% (5/21) of the papers specified on UME [42,44,47,53,55], 19% (4/21) of the papers specified on PGME [48,49,52,57], and 14% (3/21) of the papers were focused on CME [25,43,45]. Across the 21 included studies, 19 (90%) altogether described 30 current or previously offered educational programs [12,25,31-33,42-55] and 2 (10%) described elements of a curriculum framework [56,57].

### Curriculum Framework Elements

**Figure 3:**
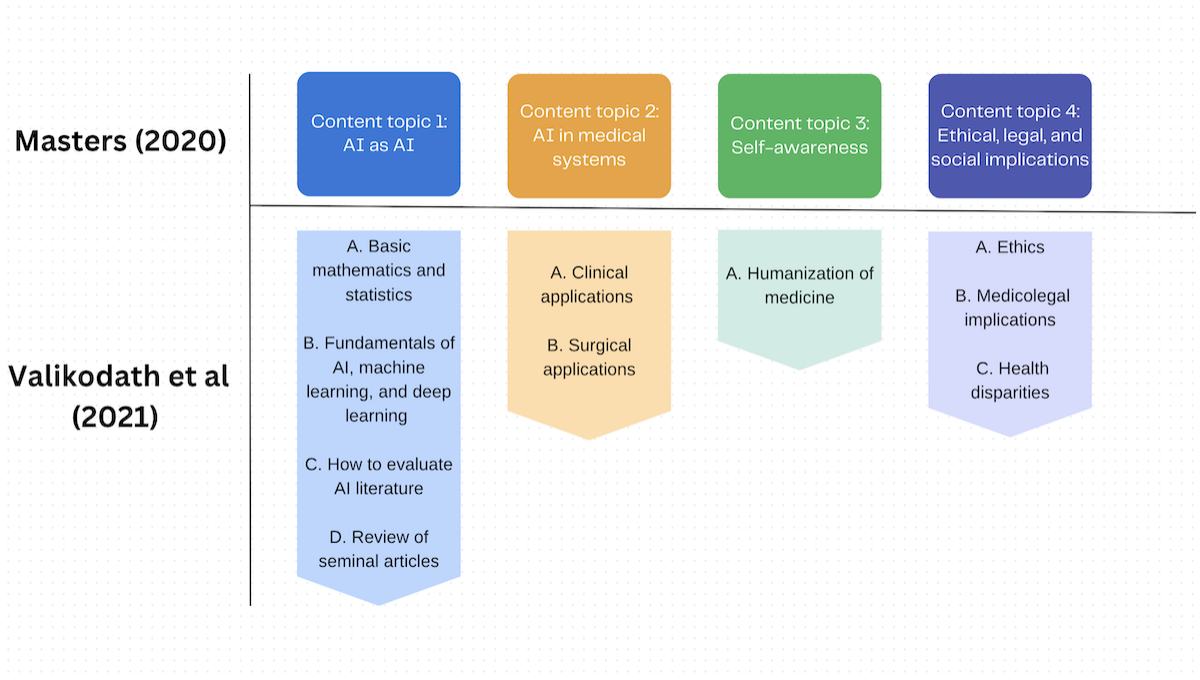
The comparison between the course content described by Masters [56] and Valikodath et al [57].

Two papers described the main elements of a curriculum framework (Table 1) [56,57]. The first paper was an opinion paper by Masters [56], which mentions 3 of the 5 elements of a curriculum framework. The paper describes the need and purpose of a curriculum, course content, and brief descriptions in terms of organization of content. The second paper to describe elements of a curriculum framework was the study by Valikodath et al [57], which provides information for all 5 elements. This includes the main purpose of an ophthalmology AI curriculum, the learning objectives, course content topics, a 4-year resident organization plan, and implementation of the curriculum, as outlined in Table 1. We noticed similarities in relation to what medical trainees should learn, as emphasized in Figure 3 [56,57].

**Table 1:** Curriculum framework studies’ characteristics (n=2).

	Masters [56]	Valikodath et al [57]
Program audience	Multiple (undergraduate medical education, PGME^a^, and continuing medical education; general)	PGME; ophthalmology
Need or purpose	This general framework will allow medical schools to assess their own position in relation to AI^b^ projects place these projects within that framework to better understand them develop new projects based on their needs	The goals of a core AI curriculum in ophthalmology include the following: recognizing major studies and discoveries of AI with regard to ophthalmology identifying the limitations of AI learning about potential applications in clinical practice
Learning objectives	—^c^	Learning objective 1: To understand the basic components of AI Learning objective 2: To identify the limitations of AI, especially in health care and research Learning objective 3: To summarize current uses of AI in ophthalmology and evaluate the primary literature Learning objective 4: To know how to potentially apply AI into clinical practice, including telemedicine and web-based visits
Course content	Topic 1. AI as AI Option A: the basics “...we need now to teach AI literacy and a basic understanding of Data Management and AI concepts, models and terminology (such as big data (and the growing number of Vs), data mining, machine learning, deep learning, supervised and unsupervised learning, natural language processing and neural networks) [...]” Option B: more advanced “...the curriculum will need to be adjusted, and electives, projects dealing with AI applications in solving medical problems, and assessing AI evaluations would be a starting point [...]” Option C: common for all “In all cases where AI is taught, the current limitations of AI need to be identified [...] Understanding these systems will be necessary to evaluate the applicability and appropriateness of solutions. [...]” Topic 2. AI in medical systems “Students will need to know the mechanics and processes of AI systems that they will be expected to use [...]” Topic 3. Self-awareness “There needs to be a self-awareness, in which the doctor is not merely using the tool, but is engaged in a cooperative exercise with the tool. This co-operation does not imply compliance, but rather operating together [...]” Topic 4. Ethical, legal, and social implications “Related to the health professionals’ perception of themselves and their role in healthcare, a host of Ethical, Legal and Social Implications emerge, and medical students will need to consider these and the questions they raise [...]”	Topic 1. Basic mathematics and statistics Topic 2. Fundamentals of AI, machine learning, deep learning Topic 3. How to evaluate AI literature Topic 4. Review of seminal articles Topic 5. Clinical applications Topic 6. Surgical applications Topic 7. Ethics Topic 8. Medicolegal implications Topic 9. Health disparities Topic 10. Humanization of medicine
Organization of content	—	Year 1 and 2: understand basic statistics and mathematics Year 1-3: become familiar with components and functions of AI Year 1-4: use web-based learning tools (articles, lectures, modules, and case-based learning) Year 2-4: assess primary literature on current AI systems in ophthalmology Year 3 and 4: understand integration of AI into clinical practice
Implementation of content	—	Teaching tools (curriculum delivery and assessment methods) background reading: articles on concepts in AI case studies web-based lecture series from experts in the field (regularly updated) interactive webinars and modules surgical simulation-based training standardized tests

^a^PGME: postgraduate medical education.

^b^AI: artificial intelligence.

^c^Not applicable.

From our comparisons, we found that the main curricular topics presented by Masters [56] appropriately corresponded to the curricular topics presented by Valikodath et al [57], for example, a main curricular topic of “AI in Medical Systems,” which describes the way in which students should learn the structures and processes of AI systems that they will be using in the future. This corresponds to “Clinical Applications” and “Surgical Applications” in which the content is targeted into learning how to use AI applications for ophthalmology. It appears that Masters’ [56] framework on course content can work as the foundation on what curricular concepts a program should include. This is because previous reviews have detailed similar curricular topics currently being taught.

### Current Educational Programs

From the 19 papers that describe an educational program, 30 current or previously offered educational programs were identified (Table 2) [12,25,31-33,42-55]. A total of 13 papers described, mentioned, or presented 24 educational programs [12,31-33,43,45-47,50-54], while 6 papers described and assessed 6 educational programs using evaluation methods (eg, survey and test scores) [25,42,44,48,49,55]. No papers described a theory, pedagogy, or framework that guided the educational program.

**Table 2:** Educational program characteristics (n=30 educational programs described in 19 papers).

Characteristic					Frequency, n (%)
**Type of educational program**					
	Course			15 (50)	
	Project			4 (13)	
	Lecture (dedicated to artificial intelligence)			4 (13)	
	Webinar			3 (10)	
	Educational summit or conference			2 (7)	
	Workshop			2 (7)	
**Pathway of education and program audience**					
	**Undergraduate medical education**			17 (57)	
		General topics	16 (94)		
		Radiology	1 (6)		
	**Postgraduate medical education**			5 (17)	
		Radiology	5 (100)		
	**Continuing medical education, n (%)**			8 (27)	
		General topics	4 (50)		
		Radiology	3 (34)		
		Cardiology	1 (13)		
**Delivery setting**					
	Medical school			23 (77)	
	National or international medical association			7 (23)	

### Educational Program Delivery

Of the 30 educational programs described collectively in the 19 remaining papers, 15 (50%) programs were courses, 4 (13%) were project-related initiatives, 4 (13%) were didactic lectures dedicated to AI, 3 (10%) were webinars, 2 (7%) were an educational summit or conference, and 2 (7%) were 1-day workshops. “AI courses were defined as elective courses focused on AI-based education. Didactic lectures dedicated to AI are 1 or 2 lectures that mention AI education but not a full course. There were 77% (23/30) educational programs delivered from a medical school, while 23% (7/30) were delivered from recognized national or international medical associations. Furthermore, it is important to clarify that some papers used multiple educational program delivery approaches. For example, an included paper explained their educational intervention was a course, but this course included didactic lectures, mentorship, and a final project. However, the reporting of this educational program’s delivery is classified as only a course and not counted as another delivery approach to minimize confusion.

Of the 30 educational programs described collectively in the 19 remaining papers, 17 (57%) UME educational programs were targeted toward medical students. Of these 17 programs, 16 (94%) were UME educational programs focused on general topics of AI in medicine and 1 (6%) was an UME educational program focused on radiology concepts. In total, 17% (5/30) of the postgraduate educational programs were for residents who were in the radiology specialty. Of the 30 educational programs, 8 (26%) were specified for practicing physicians (n=4, 50% were CME educational programs focused on general topics of AI in medicine, n=3, 37% were radiology for CME education, and n=1, 13% was in cardiology for CME). The educational program characteristics are provided in Table 2.

### Curricular Content

The following curricular concepts were adapted and framed from previous similar reviews [32,33]. The curricular content and concepts were divided into 2 types: theoretical curricular concepts and application-based curricular concepts. The subcategories and their descriptions are outlined in Table 3. The following describe the theoretical curricular concepts: fundamental of AI for using AI systems (15/19, 79%) [12,25,31-33,42-47,49,51-53]; fundamentals of health care data science for using AI systems (10/19, 53%) [12,25,31-33,45,47,49-51]; strengths and limitations of AI (9/19, 47%) [31-33,45-49]; and ethical, legal, and economic considerations of AI systems (11/19, 58%) [12,25,31-33,42,45-48,52]. The following describe the application-based curricular concepts: applications of AI systems (19/19, 100%) [12,25,31-33,42-55], operating AI systems in health care settings (10/19, 53%) [12,25,31-33,43,46,47,52,55], impact of AI on clinical reasoning and medical decision-making (7/19, 37%) [12,25,31-33,43,55], communication of AI results to patients (4/19, 21%) [12,31-33], and critical appraisal of AI systems (7/19, 37%) [12,31-33,50,53,54].

**Table 3:** Curricular concepts mentioned in the educational program papers (n=19).

AI^a^ curricular concept		Description of curricular concept	Reference
**Theoretical curricular concepts (learning what is AI in medicine)^b^**			
	Fundamental of AI for using AI systems	Providing an overview of AI definitions and concepts, including machine learning; natural language processing; and the basics of data acquisition, cleaning, analysis, and visualization	[12,25,31-33,42-47,49,51-53]
	Fundamentals of health care data science for using AI systems	Providing an overview of the environment supporting AI, which includes biostatistics, big data, and the use and processing of data by algorithms and machine learning	[12,25,31-33,45,47,49-51]
	Strengths and limitations of AI	Promoting learners’ comprehension of the advantages and limitations of various AI systems, such as factors that affect AI accuracy (eg, sources of error and bias)	[31-33,45-49]
	Ethical, legal, and economic considerations of AI systems	Developing a comprehensive understanding of ethics, equity, inclusion, patient rights, and confidentiality, alongside regulatory frameworks, policy considerations, liability, and intellectual property issues related to using AI systems as well as grasping the potential alterations to business or clinical processes resulting from the integration of AI technologies	[12,25,31-33,42,45-48,52]
**Application-based curricular concepts (learning how to use AI for clinical practice)^c^**			
	Applications of AI systems	Familiarizing with clinical application of AI systems in clinical practice to understand how they are used	[12,25,31-33,42-55]
	Operating AI systems in health care settings	Understanding how to embed and engage with AI tools into clinical settings and workflows (eg, learning to engage in data mining tools or how to properly communicate with AI systems to receive meaningful results)	[12,25,31-33,43,46,47,52,55]
	Impact of AI on clinical reasoning and medical decision-making	Having the ability to understand, interpret, and apply results of AI systems in clinical practice	[12,25,31-33,43,55]
	Communication of AI results to patients	Communicate findings to patients in a personalized and meaningful manner and engage in discussions regarding the use of AI in the medical decision-making process	[12,31-33]
	Critical appraisal of AI systems	Acquiring proficiency in assessing diagnostic and therapeutic algorithms powered by AI to ensure safe and effective integration and use in clinical practice	[12,31-33,50,53,54]

^a^AI: artificial intelligence.

^b^The mentioned concepts encompass foundational learning that serves as the basis of medical artificial intelligence educational philosophy and clinical practice.

^c^The mentioned concepts prioritize the practical applications of artificial intelligence knowledge and skills in a clinical context.

### Assessment of Educational Outcomes

Of the 19 papers, 6 (32%) presented the results of their evaluation of an educational program (Table 4) [25,42,44,48,49,55]. Two papers described only level 1 evaluation outcomes (eg, learner reaction and satisfaction with the educational program) in which participants were overall very satisfied with the AI content learned [42,48]. Four papers described level 2 evaluation outcomes (eg, change in attitude, knowledge, or skill) in which learners demonstrated acquisition of a variety of competencies (linear algebra pertaining to AI and basics of AI) and skills (eg, incorporate medical decisions given by an algorithm and implementing AI in clinical practice) [25,44,49,55] where two of these papers also evaluated level 1 outcomes [25,49]. There were no outcomes that could be categorized as level 3 or level 4; thus, the program evaluations did not comment on the change in behavior or affect at the organizational level or on patient outcomes.

**Table 4:** Studies describing evaluation outcomes (n=6).

Study	Educational program	Levels and outcomes of the model of training evaluation developed by Kirkpatrick and Kirkpatrick [39]
Alderson et al [42], 2021	Course	Level 1: “...satisfaction scores of 4.4/5.0 (n=13) [...]”
Barbour et al [44], 2019	Educational summit	Level 2: “...there was a general belief [about 70% from the figures] that AI would make health care less humanistic.” Level 2: “...did not observe a meaningful shift in attitudes regarding the desire to take a leadership role in developing or implementing AI [...]” Level 2: “Attendees arrived believing they had a poor baseline understanding of AI’s role in health care, and left the summit with an enhanced understanding of the topic [...]”
Hedderich et al [25], 2021	Course	Level 1: “The participants were overall very satisfied with the study material and the organization of the course, and deemed the content of the course important for their work as a clinician or scientist.” Level 2: “...self-perceived skills improved in all areas, for understanding Python code as well as for understanding concepts of linear algebra pertaining to AI.” Level 2: “...participants felt more confident to analyze a research paper in the field, to implement an AI algorithm in a clinical environment, and to incorporate the decisions given by an algorithm into their clinical decision making.” Level 2: “Most of the participants felt more competent at dealing with AI in medical imaging after the course.”
Kang et al [48], 2017	Workshop	Level 1: “Ninety percent of the residents... reported that the course was helpful or very helpful […]” Level 1: “...94% of the participants...felt that the lectures were of high or very high quality.” Level 1: “Eighty-two percent...reported that they planned to pursue additional educational or research training in CER or big data analytics after the course [...]” Level 1: “[...] 98% of the respondents felt that health services and big data research are important or very important for the future of radiology.’
Lindqwister et al [49], 2021	Course	Level 1: “Exit surveys demonstrated a high degree of learner satisfaction, with an aggregate rating of 9.8/10.” Level 2: “There is a statistically significant difference between all pre- and postlecture question results (*P*<.04) by Wilcoxon Sign-rank test.”
Tschirhart et al [55], 2022	Workshop	Level 2: “...considerable improvement in the first independent dataset, with further improvement in subsequent datasets [...]”

## Discussion

### Principal Findings

The development and implementation of AI in medical education has greatly increased within the last decade, specifically since the COVID-19 pandemic where there was a global shift into the digital world accelerating the development of AI technology [59]. This can be seen as the majority (15/21, 77%) of included papers within this review were published since COVID-19 pandemic. Although there is a growing field within research and practice, AI medical education, specifically within curricula development, is still limited. We found that the current curriculum frameworks for AI medical education are limited, indicating a need for further research. We also found that the current state of AI educational programs lack the use of a theory, framework, or pedagogy. In addition, we uncovered alternative methods and different levels of in-depth curriculum planning for AI in medical education.

### Current State of Curriculum Frameworks for AI Medical Education

This is the first review to identify curriculum frameworks for AI medical education, and our findings demonstrate that they are very limited. Although the literature is abundant in terms of recommendations and potential plans of actions for integrating AI education within medical education, there is an inadequate amount of formal curricula or frameworks [20,60,61]. For example, curricular recommendations lack specific learning outcomes and are not based on a particular education theory, as they usually focus solely on the content or competencies that should be taught [32,56]. Although understanding what concepts should be taught in AI is important, curriculum frameworks must be as comprehensive as possible.

From the identified frameworks, Masters [56] outlines a broad framework for any level of education, while Valikodath et al [57] outlines a complete framework for ophthalmology residency education. Their frameworks remain dissimilar in all aspects, except in how their course content was described. As seen with these 2 papers, the lack of curriculum frameworks in the literature is staggering. Further studies should focus on the development of these frameworks and start thinking on how to plan for the impending changes in medical education. As Valikodath et al [57] demonstrated their AI curriculum framework for ophthalmology, other specialties should follow suit, as AI affects each specialty differently [9]. Overall, the current state of curriculum framework in medical education appears to be far from sufficient in the existing literature, and further research is needed.

### Current State of AI Medical Educational Programs

#### Overview

In comparison to curriculum frameworks, educational programs in this field have been reviewed recently, specifically in the past 3 years [31-33]. However, research in AI medical education evolves quickly, and thus, a further identification of programs was carried out. We specifically looked at the framework, pedagogy, or learning theory described; the content and its audience; and if the program was evaluated for outcomes, which were used to assess its effectiveness, according to the model developed by Kirkpatrick et al [39].

#### The Lack of Learning Theories and Pedagogies

There were no papers that referenced a framework, pedagogy, or learning theory that guided the existence of the educational program. However, the use of frameworks, pedagogies, or learning theories is important for informing the development of valid, accurate, and competent educational programs [62-64]. By using frameworks, pedagogies, or learning theories, educators can choose the most effective instructional tactics, learning objectives, assessment, and evaluation approaches that can best help their students to learn [65]. A recent paper that fell outside the scope of our search date describes the use of constructivist theory and backward design learning principles that guided the development of their AI course [66]. Further papers should implement and report on a learning theory, framework, or pedagogy, as they have a role in medical education [65].

#### The Generalized AI Medical Content

The integration of AI concepts and topics within medical education remains generalized throughout the different levels of medical education, as seen with the educational programs described in our review. A total of 20 educational programs were described as focusing on general topics such as introductions to AI or information on AI and its application to medicine. The only postgraduate and continuing educational programs that had an AI-specific educational material were radiology, ophthalmology, and cardiology. This can be attributed to various reasons, including the constant evolution and novelty of AI technology, which may describe why generalized educational programs for AI appear across the medical educational continuum [67]. Radiology had the highest number of educational programs and was seen in all levels of medical education because AI in medicine was first applied in the field of radiology as it detected microcalcifications in a mammography during the year of 1992, or it could be due to the field being highly technological [68]. It is encouraging to see that specialties such as ophthalmology and cardiology have increased interest in AI education; other specialties and medical institutions should begin to follow suit. This is encouraging as it demonstrates that other specialties besides the highly technological field of radiology have been learning AI within medical education. This is especially important as more fields of medicine besides radiology are integrating AI within their practice, such as cardiology, pathology, and ophthalmology [3]. Furthermore, most of the educational programs were found in UME and within medical schools, which is ideal as it introduces a large audience of medical students to the concept of AI and its applications early in their careers.

#### The Success of Current AI Educational Programs

The included literature demonstrates that current efforts are being made to evaluate the outcomes of AI-related educational initiatives. According to the model developed by Kirkpatrick et al [39], an internationally recognized tool for evaluating and analyzing the results of educational, training, and learning programs, current AI programs have overall been positively received by medical learners. This was represented by the positive reactions, opinions, and attitudes toward AI after completing an educational program (level 1) as well as the acquisition of AI-related knowledge, skills, and confidence (level 2). These findings were also presented in a similar review in which the AI educational programs they identified also had positive outcomes, which were categorized as level 1 or level 2 [33]. However, further studies must assess educational programs for outcomes in relation to behavioral changes (level 3), specifically if there has been a transfer of AI-related knowledge, skills, and abilities into their daily work.

Further studies should also assess how the acquisition and application of these AI-related knowledge, skills, and abilities has affected the organization as a whole (eg, Has the increase in AI-educated physicians improved overall efficiency at the hospital?) or on patient outcomes (eg, Has there been an improvement in the patient’s functional status or safety because of AI-educated physicians? [level 4]). By assessing for these additional outcomes, educators and medical organizations can understand how current AI educational programs have affected physician performance with AI technology. Increased research on the evaluations of educational programs can help further validate current educational tools and be used as inspiration for other institutions to create their own educational material. As seen in the review [33], there is a lack of consistency in the measures of these outcomes, as self-constructed and nonvalidated instruments were also used. Future studies should develop a validated tool to evaluate educational outcomes for a comprehensive synthesis.

### Curriculum Planning and Framework Development of AI Medical Education

Curriculum planning of AI educational initiatives within medical education is insufficient. Although limited studies of curriculum frameworks were published, other forms of curriculum planning can be seen in the literature. Some medical institutions have conducted AI perception surveys [69,70], curriculum needs assessment surveys [71-73], and an interview [74] to understand what should be integrated into the AI medical curriculum. These studies are promising and contribute to the overall efforts to understanding how current educators, medical students, residents, and physicians consider AI within their educational system.

The absence of curriculum frameworks is staggering, especially given that AI competence is likely to become a required skill for medical graduates [75]. The development of AI curricula and frameworks have already been gaining traction across other fields of education and levels. This can be seen as early as childhood education; for example, Su and Zhong [76] present their own curriculum framework, which outlines their concepts, teaching methods, teaching activities, projects, and assessment suggestions for AI education.

From a global perspective, the United Nations Educational, Scientific, and Cultural Organization, a specialized agency of the United Nations, released a document outlining the current practices of developing and implementing AI curricula in primary and secondary school education (K-12) [77]. From their report, several types of frameworks for AI literacy have been suggested, such as the AI Literacy Competency Framework, the AI4K12: 5 Big Ideas Framework, and the Machine Learning Education Framework. These recent reports and papers suggest increased efforts to integrate AI education before postsecondary school, which further stresses the importance of developing AI curricula and frameworks in medical education. Although there are current educational frameworks for AI education, each target audience must have their own specialized curricula to tailor the educational needs of the learners.

Medical educators can develop their curriculum through several different methodologies, such as the 10 key questions to be addressed while developing a curriculum [78] and the 6-step approach for curricular design [79]. However, curriculum frameworks allow a visual and detailed road map to implement a curriculum. Through this detailed format, educators are able to easily navigate the curriculum and its implementation, especially for new concepts in medicine, such as AI. To develop curriculum frameworks for AI in medicine, there must be an interdisciplinary team consisting of medical educators, AI experts and users, researchers, and curriculum designers due to the multiple fields incorporated.

The introduction of AI in medicine must be properly structured and organized within UME, PGME, and CME. Therefore, curriculum frameworks should be properly established through different levels of education and specialties. This has been emphasized by other reviews that call for integration of AI education in all levels and, thus, all specialties of medicine [17,33]. For example, a curriculum framework for UME will be different than a curriculum framework for PGME in dermatology. Curriculum frameworks can be adapted and they most likely will be, especially since AI education in medical education is still in its infancy. This is where leaders in UME, PGME, and CME organizations (eg, policy makers, medical educators, and researchers) must communicate effectively to eliminate any crossover education and repeated information. New technology and innovations in relation to AI and medicine will inevitably occur; however, it is important to be cognizant of the fundamentals of AI and how it will affect a physician’s practice at the time. Sufficient planning of an AI curricula will deliver effective education for physicians who will increasingly be using AI technology in the near future; therefore, medical educators and institutions must begin to consider curriculum planning.

### Incorporating and Advocating for AI Into the Medical Curriculum

The literature emphasizes the need to introduce AI in the medical education curriculum [12,13,15-20]; however, there are several challenges that have been discussed in terms of implementing this type of education. This includes insufficient time, insufficient resources (eg, lack of teaching staff or knowledge), and variable aptitude and interest in AI [80-82]. However, this review details several approaches to implementation as well as 6 studies that have evaluated their educational program. These successful educational programs can provide medical schools and national and international medical organizations with examples of current AI content topics and implementation methods that have worked for others. These medical education institutions can view how AI-based medical education is currently being offered around the world and understand any challenges, opportunities, and strengths about these programs. Although the content and provision of AI education is heterogenous, this heterogeneity can allow educators and students to view the many types of programs that were offered. As AI education for medicine is still in its infancy, educators should explore these programs where they can then potentially modify an educational program that best suits their needs. As seen in this review, there are several ways to incorporate AI material into the current curriculum seamlessly, such as an AI fundamentals lecture or module, an AI elective, or a research project.

Medical students, residents, and practicing physicians also have the opportunity to advocate for the inclusion of AI education at their respective institutions [46]. For example, there are several North American university chapters of the Artificial Intelligence in Medicine Student Society, such as the University of Toronto and University of Alberta, which organizes workshops, conferences, and multiple speaker sessions throughout the year [46]. These student interest groups demonstrate the increased interest for AI and can potentially build momentum and advocate for AI education at their respective institutions. As some of the offerings at these student interest groups include brief educational material for AI, medical institutions can work with these students as a starting point.

### Strengths and Limitations

The strengths of this review include the comprehensive search strategies, the inclusion of a variety of information sources, and rigorous methodological approaches that are replicable. For example, study selection was completed by 2 reviewers, and disagreements were resolved by discussion or consensus involving a third investigator. Furthermore, a scoping review protocol was registered and published to improve transparency of the methodological process.

Although this study was conducted in a structured and systematic manner, there are some limitations that are important to consider. A limited number of papers were retrieved during the search and selection process. Only 2 papers reported having a curriculum framework, with 1 reporting a full curricula plan related to AI in medicine. This can be because AI technology is emerging and continuing to change within medicine and it has been limiting in terms of educational advances. Because of the nature of the scoping review, the quality of each identified study was not assessed.

### Conclusions

Medicine is rapidly evolving from the information age to the age of AI, where machines will become an integral part of medical practice. Thus, medical education needs to keep pace with changes in medical practice. This review synthesized knowledge from the literature on curriculum frameworks and current educational programs that focus on the teaching and learning of AI for medical students, residents, and practicing physicians. To better integrate AI curricula into the continuum of medical education, discussions surrounding curriculum planning of AI should begin where institutions are recommended to work collaboratively with teams of curriculum designers, data scientists, and medical educators to develop AI curricula and educational programs. There is a need to (1) develop a general AI education curriculum framework for UME; (2) develop a specific AI education curriculum framework for each specialty within PGME and CME; and (3) design, implement, and evaluate current educational programs. Overall, institutions must begin equipping current and future physicians with the knowledge, skills, and confidence to effectively use AI applications as it will continue to grow within the field of health care.

## Appendix

Multimedia Appendix 1 PRISMA-ScR (Preferred Reporting Items for Systematic reviews and Meta-Analyses extension for Scoping Reviews) checklist.

Multimedia Appendix 2 Study characteristics (N=21).

Multimedia Appendix 3 Countries and years of publications included in the review.

## Abbreviations

AI: artificial intelligence
CME: continuing medical education
PGME: postgraduate medical education
PRISMA: Preferred Reporting Items for Systematic Reviews and Meta-Analyses
PRISMA-ScR: Preferred Reporting Items for Systematic Reviews and Meta-Analyses extension for Scoping Reviews
UME: undergraduate medical education

## References

[1] Davenport T, Kalakota R (2019). The potential for artificial intelligence in healthcare. Future Healthc J.

[2] Chan KS, Zary N (2019). Applications and challenges of implementing artificial intelligence in medical education: integrative review. JMIR Med Educ.

[3] Ahuja AS (2019). The impact of artificial intelligence in medicine on the future role of the physician. PeerJ.

[4] Hosny A, Parmar C, Quackenbush J, Schwartz LH, Aerts HJ (2018). Artificial intelligence in radiology. Nat Rev Cancer.

[5] Liu Y, Kohlberger T, Norouzi M, Dahl GE, Smith JL, Mohtashamian A, Olson N, Peng LH, Hipp JD, Stumpe MC (2019). Artificial intelligence–based breast cancer nodal metastasis detection: insights into the black box for pathologists. Arch Path Lab Med.

[6] Esteva A, Kuprel B, Novoa RA, Ko J, Swetter SM, Blau HM, Thrun S (2017). Dermatologist-level classification of skin cancer with deep neural networks. Nature.

[7] Abbasgholizadeh Rahimi S, Légaré F, Sharma G, Archambault P, Zomahoun HT, Chandavong S, Rheault N, T Wong S, Langlois L, Couturier Y, Salmeron JL, Gagnon M, Légaré J (2021). Application of artificial intelligence in community-based primary health care: systematic scoping review and critical appraisal. J Med Internet Res.

[8] Birkhoff DC, van Dalen AS, Schijven MP (2021). A review on the current applications of artificial intelligence in the operating room. Surg Innov.

[9] Topol EJ (2019). High-performance medicine: the convergence of human and artificial intelligence. Nat Med.

[10] Paul D, Sanap G, Shenoy S, Kalyane D, Kalia K, Tekade RK (2021). Artificial intelligence in drug discovery and development. Drug Discov Today.

[11] Han ER, Yeo S, Kim MJ, Lee YH, Park KH, Roh H (2019). Medical education trends for future physicians in the era of advanced technology and artificial intelligence: an integrative review. BMC Med Educ.

[12] Paranjape K, Schinkel M, Nannan Panday R, Car J, Nanayakkara P (2019). Introducing artificial intelligence training in medical education. JMIR Med Educ.

[13] Wartman SA, Combs CD (2018). Medical education must move from the information age to the age of artificial intelligence. Acad Med.

[14] Minor LB (2020). Stanford medicine 2020 health trends report: the rise of the data-driven physician. Stanford Medicine.

[15] Pucchio A, Papa JD, de Moraes FY (2022). Artificial intelligence in the medical profession: ready or not, here AI comes. Clinics (Sao Paulo).

[16] Kolachalama VB, Garg PS (2018). Machine learning and medical education. NPJ Digit Med.

[17] Mehta S, Vieira D, Quintero S, Bou Daher D, Duka F, Franca H, Bonilla J, Molnar A, Molnar C, Zerpa D, Fleming Díaz MF (2020). Redefining medical education by boosting curriculum with artificial intelligence knowledge. J Cardiol Curr Res.

[18] Abdulhussein H, Turnbull R, Dodkin L, Mitchell P (2021). Towards a national capability framework for artificial intelligence and digital medicine tools – a learning needs approach. Intell Based Med.

[19] James CA, Wheelock KM, Woolliscroft JO (2021). Machine learning: the next paradigm shift in medical education. Acad Med.

[20] Lomis K, Jeffries P, Palatta A, Sage M, Sheikh J, Sheperis C, Whelan A (2021). Artificial intelligence for health professions educators. NAM Perspect.

[21] Topol E (2019). The Topol review: preparing the health care work- force to deliver the digital future. National Health Service, UK.

[22] (2018). AMA passes first policy recommendations on augmented intelligence internet. American Medical Association.

[23] Reznick RK, Harris K, Horsley T, Hassani MS Artificial intelligence (AI) and emerging digital technologies. The Royal College of Physicians and Surgeons of Canada.

[24] Pinto Dos Santos D, Giese D, Brodehl S, Chon SH, Staab W, Kleinert R, Maintz D, Baeßler B (2019). Medical students' attitude towards artificial intelligence: a multicentre survey. Eur Radiol.

[25] Hedderich DM, Keicher M, Wiestler B, Gruber MJ, Burwinkel H, Hinterwimmer F, Czempiel T, Spiro JE, Pinto Dos Santos D, Heim D, Zimmer C, Rückert D, Kirschke JS, Navab N (2021). AI for doctors-a course to educate medical professionals in artificial intelligence for medical imaging. Healthcare (Basel).

[26] Stabback P (2007). Guidelines for constructing a curriculum framework for basic education. International Bureau of Education, UNESCO.

[27] Redwood-Campbell L, Pakes B, Rouleau K, MacDonald CJ, Arya N, Purkey E, Schultz K, Dhatt R, Wilson B, Hadi A, Pottie K (2011). Developing a curriculum framework for global health in family medicine: emerging principles, competencies, and educational approaches. BMC Med Educ.

[28] Rampton V, Mittelman M, Goldhahn J (2020). Implications of artificial intelligence for medical education. Lancet Digit Health.

[29] Obadeji A (2019). Health professions education in the 21st century: a contextual curriculum framework for analysis and development. J Contemp Med Edu.

[30] Iqbal S, Ahmad S, Akkour K, Wafa AN, AlMutairi HM, Aldhufairi AM (2021). Review article: impact of artificial intelligence in medical education. MedEdPublish.

[31] Grunhut J, Wyatt AT, Marques O (2021). Educating future physicians in artificial intelligence (AI): an integrative review and proposed changes. J Med Educ Curric Dev.

[32] Lee J, Wu AS, Li D, Kulasegaram KM (2021). Artificial intelligence in undergraduate medical education: a scoping review. Acad Med.

[33] Charow R, Jeyakumar T, Younus S, Dolatabadi E, Salhia M, Al-Mouaswas D, Anderson M, Balakumar S, Clare M, Dhalla A, Gillan C, Haghzare S, Jackson E, Lalani N, Mattson J, Peteanu W, Tripp T, Waldorf J, Williams S, Tavares W, Wiljer D (2021). Artificial intelligence education programs for health care professionals: scoping review. JMIR Med Educ.

[34] Peters MD, Godfrey C, McInerney P, Munn Z, Tricco AC, Khalil H, Aromataris E, Lockwood C, Porritt K, Pilla B, Jordan Z (2010). Scoping reviews. JBI Manual for Evidence Synthesis.

[35] Arksey H, O'Malley L (2005). Scoping studies: towards a methodological framework. Int J Soc Res Methodol.

[36] Levac D, Colquhoun H, O'Brien KK (2010). Scoping studies: advancing the methodology. Implement Sci.

[37] Tricco AC, Lillie E, Zarin W, O'Brien KK, Colquhoun H, Levac D, Moher D, Peters MD, Horsley T, Weeks L, Hempel S, Akl EA, Chang C, McGowan J, Stewart L, Hartling L, Aldcroft A, Wilson MG, Garritty C, Lewin S, Godfrey CM, Macdonald MT, Langlois EV, Soares-Weiser K, Moriarty J, Clifford T, Tunçalp Ö, Straus SE (2018). PRISMA extension for scoping reviews (PRISMA-ScR): checklist and explanation. Ann Intern Med.

[38] Tolentino R, Baradaran A, Gore G, Pluye P, Abbasgholizadeh-Rahimi S (2023). Curriculum frameworks and educational programs in artificial intelligence for medical students, residents, and practicing physicians: a scoping review protocol. JBI Evid Synth.

[39] Kirkpatrick DL, Kirkpatrick JD (2006). Evaluating Training Programs: The Four Levels.

[40] Kaul V, Enslin S, Gross SA (2020). History of artificial intelligence in medicine. Gastrointest Endosc.

[41] Popay J, Roberts H, Sowden A, Petticrew M, Arai L, Rodgers M, Britten N, Roen K, Duffy S (2006). Guidance on the conduct of narrative synthesis in systematic reviews: a product from the ESRC methods programme version. Lancaster University.

[42] Alderson PO, Donlin MJ, Morrison LA A model to introduce medical students to the use of artificial intelligence and genomics for precision medicine. medRxiv.

[43] Balthazar P, Tajmir SH, Ortiz DA, Herse CC, Shea LA, Seals KF, Cohen-Addad D, Purkayastha S, Gichoya JW (2020). The artificial intelligence journal club (#RADAIJC): a multi-institutional resident-driven web-based educational initiative. Acad Radiol.

[44] Barbour AB, Frush JM, Gatta LA, McManigle WC, Keah NM, Bejarano-Pineda L, Guerrero EM (2019). Artificial intelligence in health care: insights from an educational forum. J Med Educ Curric Dev.

[45] Forney MC, McBride AF (2020). Artificial intelligence in radiology residency training. Semin Musculoskelet Radiol.

[46] Harish V, Bilimoria K, Mehta N, Morgado F, Aissiou A, Eaton S, Hu S, Ji F, Lia H, MacMillan K, McLeod G (2019). Preparing medical students for the impact of artificial intelligence on healthcare. Canadian Federation of Medical Students.

[47] Hu R, Fan KY, Pandey P, Hu Z, Yau O, Teng M, Wang P, Li T, Ashraf M, Singla R (2022). Insights from teaching artificial intelligence to medical students in Canada. Commun Med (Lond).

[48] Kang SK, Lee CI, Pandharipande PV, Sanelli PC, Recht MP (2017). Residents' introduction to comparative effectiveness research and big data analytics. J Am Coll Radiol.

[49] Lindqwister AL, Hassanpour S, Lewis PJ, Sin JM (2021). AI-RADS: an artificial intelligence curriculum for residents. Acad Radiol.

[50] McCoy LG, Nagaraj S, Morgado F, Harish V, Das S, Celi LA (2020). What do medical students actually need to know about artificial intelligence?. NPJ Digit Med.

[51] Nagy M, Radakovich N, Nazha A (2022). Why machine learning should be taught in medical schools. Med Sci Educ.

[52] Nguyen GK, Shetty AS (2018). Artificial intelligence and machine learning: opportunities for radiologists in training. J Am Coll Radiol.

[53] Park SH, Do KH, Kim S, Park JH, Lim YS (2019). What should medical students know about artificial intelligence in medicine?. J Educ Eval Health Prof.

[54] Sapci AH, Sapci HA (2020). Artificial intelligence education and tools for medical and health informatics students: systematic review. JMIR Med Educ.

[55] Tschirhart J, Woolsey A, Skinner J, Ahmed K, Fleming C, Kim J, Dave C, Arntfield R (2023). Introducing medical students to deep learning through image labelling: a new approach to meet calls for greater artificial intelligence fluency among medical trainees. Can Med Educ J.

[56] Masters K (2020). Artificial intelligence developments in medical education: a conceptual and practical framework. MedEdPublish (2016).

[57] Valikodath NG, Cole E, Ting DS, Campbell JP, Pasquale LR, Chiang MF, Chan RV, American Academy of Ophthalmology Task Force on Artificial Intelligence (2021). Impact of artificial intelligence on medical education in ophthalmology. Transl Vis Sci Technol.

[58] Page MJ, McKenzie JE, Bossuyt PM, Boutron I, Hoffmann TC, Mulrow CD, Shamseer L, Tetzlaff JM, Akl EA, Brennan SE, Chou R, Glanville J, Grimshaw JM, Hróbjartsson A, Lalu MM, Li T, Loder EW, Mayo-Wilson E, McDonald S, McGuinness LA, Stewart LA, Thomas J, Tricco AC, Welch VA, Whiting P, Moher D (2021). The PRISMA 2020 statement: an updated guideline for reporting systematic reviews. BMJ.

[59] Sun L, Yin C, Xu Q, Zhao W (2023). Artificial intelligence for healthcare and medical education: a systematic review. Am J Transl Res.

[60] Nagy M, Radakovich N, Nazha A (2020). Machine learning in oncology: what should clinicians know?. JCO Clin Cancer Inform.

[61] Ngo B, Nguyen D, van Sonnenberg E (2021). Artificial intelligence: has its time come for inclusion in medical school education? Maybe…maybe not. MedEdPublish.

[62] Tredinnick-Rowe J, Cavero OB, Llevot-Calvet N (2018). The role of pedagogy in clinical education. New Pedagogical Challenges in the 21st Century - Contributions of Research in Education.

[63] Khalil MK, Elkhider IA (2016). Applying learning theories and instructional design models for effective instruction. Adv Physiol Educ.

[64] Fuller JC, Woods ME (2021). The science of learning: why learning theories matter in graduate medical education. HCA Healthc J Med.

[65] Mukhalalati BA, Taylor A (2019). Adult learning theories in context: a quick guide for healthcare professional educators. J Med Educ Curric Dev.

[66] Krive J, Isola M, Chang L, Patel T, Anderson M, Sreedhar R (2023). Grounded in reality: artificial intelligence in medical education. JAMIA Open.

[67] Grassini S (2023). Shaping the future of education: exploring the potential and consequences of AI and ChatGPT in educational settings. Educ Sci.

[68] Driver CN, Bowles BS, Bartholmai BJ, Greenberg-Worisek AJ (2020). Artificial intelligence in radiology: a call for thoughtful application. Clin Transl Sci.

[69] Mehta N, Harish V, Bilimoria K, Morgado F, Ginsburg S, Law M, Das S (2021). Knowledge and attitudes on artificial intelligence in healthcare: a provincial survey study of medical students. MedEdPublish.

[70] Wood EA, Ange BL, Miller DD (2021). Are we ready to integrate artificial intelligence literacy into medical school curriculum: students and faculty survey. J Med Educ Curric Dev.

[71] Civaner MM, Uncu Y, Bulut F, Chalil EG, Tatli A (2022). Artificial intelligence in medical education: a cross-sectional needs assessment. BMC Med Educ.

[72] Gray K, Slavotinek J, Dimaguila GL, Choo D (2022). Artificial intelligence education for the health workforce: expert survey of approaches and needs. JMIR Med Educ.

[73] Ejaz H, McGrath H, Wong BL, Guise A, Vercauteren T, Shapey J (2022). Artificial intelligence and medical education: a global mixed-methods study of medical students' perspectives. Digit Health.

[74] Weidener L, Fischer M (2023). Artificial intelligence teaching as part of medical education: qualitative analysis of expert interviews. JMIR Med Educ.

[75] Çalışkan SA, Demir K, Karaca O (2022). Artificial intelligence in medical education curriculum: an e-Delphi study for competencies. PLoS One.

[76] Su J, Zhong Y (2022). Artificial Intelligence (AI) in early childhood education: curriculum design and future directions. Comput Educ Artif Intell.

[77] Miao F, Shiohira K K-12 AI curricula: a mapping of government-endorsed AI curricula. United Nations Educational, Scientific and Cultural Organization.

[78] Harden RM (1986). Ten questions to ask when planning a course or curriculum. Med Educ.

[79] Thomas PA, Kern DE, Hughes MT, Tackett SA, Chen BY (2022). Curriculum Development for Medical Education – A Six–Step Approach.

[80] Azer SA, Guerrero AP (2023). The challenges imposed by artificial intelligence: are we ready in medical education?. BMC Med Educ.

[81] Grunhut J, Marques O, Wyatt AT (2022). Needs, challenges, and applications of artificial intelligence in medical education curriculum. JMIR Med Educ.

[82] Ng FY, Thirunavukarasu AJ, Cheng H, Tan TF, Gutierrez L, Lan Y, Ong JC, Chong YS, Ngiam KY, Ho D, Wong TY, Kwek K, Doshi-Velez F, Lucey C, Coffman T, Ting DS (2023). Artificial intelligence education: an evidence-based medicine approach for consumers, translators, and developers. Cell Rep Med.

