# Supplementary material for: Curriculum Frameworks and Educational Programs in AI for Medical Students, Residents, and Practicing Physicians: Scoping Review
Source: JMIR Med Educ. 2024 Jul 18;10:e54793. doi: 10.2196/54793 (PMC11294785; doi:10.2196/54793)
Supplement: Multimedia Appendix 2 [file mededu_v10i1e54793_app2.docx]

**Multimedia Appendix 2.** Study characteristics (N=21)

| **Author (Year), Country** | **Type of Paper** | **Aim of Paper** | **Setting** | **Curriculum Framework or Educational Program** |
| --- | --- | --- | --- | --- |
| Alderson et al. (2021) [42], USA | Empirical (cross-sectional survey) | “[…] sought to introduce pre-clinical students to the importance of AI methodologies and medical applications using modular short courses focused on active learning with precision medicine as a primary use case.” | UME; precision medicine | Educational Program |
| Balthazar et al. (2020) [43], USA | Perspective | “[…] describe one such initiative [AI Journal Club] led by radiology residents in collaboration with the American College of Radiology […] discussing the role of Radiologists as Knowledge Experts in a world of Artificial Intelligence.” | CME; radiology | Educational Program |
| Barbour et al. (2019) [44], USA | Commentary | The commentary describes lessons learned during the development and implementation of an educational program (summit) discussing on AI in healthcare. | UME; general | Educational Program |
| Charow et al. (2021) [33], Canada | Review | “[…] provides an overview of the types of current or past AI education programs that pertains to the programs’ curricular content, modes of delivery, critical implementation factors for education delivery, and outcomes used to assess the programs’ effectiveness.” | Multiple (UME, PGME, CME; multiple) | Educational Program |
| Forney & McBride (2020) [45], USA | Review | “[…] [describes that residents should have] an understanding of the fundamentals and types of AI in radiology, the broad areas AI can be applied in radiology, how to assess AI applications in radiology, and resources available to build their knowledge in AI applications in radiology.” | CME; radiology | Educational Program |
| Grunhut et al. (2021) [31], USA | Review | "[…] review the current literature that covers the attitudes of medical students towards AI, implementation of AI in the medical curriculum, and describe the need for more research in this area.” | Multiple (UME, PGME, CME; multiple) | Educational Program |
| Harish et al. (2019) [46], Canada | Position paper | “[…] outline recommendations to develop AI learning objectives aligned with the core CanMEDS roles of Advocate, Leader, and Medical Expert, equipping medical students with the necessary competencies to navigate the health care environments of tomorrow.” | Multiple (UME; radiology, UME; general) | Educational Program |
| Hedderich et al. (2021) [25], Germany | Empirical (cross-sectional survey) | “[…] report on [an] initial experience with [an] educational program and how the participants perceived it […] [and] assessed the participants’ opinions on AI in medical imaging, as well as their self-rated skills pertaining to the topic in order to inform other institutions seeking to develop educational programs for [medical doctors] in medical imaging.” | CME; medical imaging | Educational Program |
| Hu et al. (2022) [47], Canada | Commentary | “[…] describe an AI training curriculum that was developed and delivered to Canadian medical undergraduates and provide recommendations for future training.” | UME; general | Educational Program |
| Kang et al. (2017) [48], USA | Opinion | “[…] describe [their] work to organize and present the mini-course to residents […] and summarize survey responses gathered to date from the participants about their experiences in the course.” | PGME; radiology | Educational Program |
| Lee et al. (2021) [32], USA | Review | “ […] to identify gaps and key themes in the peer-reviewed literature on AI training in [Undergraduate Medical Education].” | Multiple (UME, PGME, CME; multiple) | Educational Program |
| Lindqwister et al. (2021) [49], Canada | Empirical (cross-sectional survey) | “[…] presents [an] institution’s efforts to address [the rise of AI within radiology] […] as a model for a successful introductory curriculum into artificial intelligence in radiology titled AI-RADS.” | PGME; radiology | Educational Program |
| Masters (2020) [56], Oman | Opinion | “[…] provide an AI conceptual and practical framework for medical education administrators and educators, so that they may have a clearer understanding of the current situation, and may be better placed to guide future AI developments to meet their needs in medical education.” | Multiple (UME, PGME, CME; general) | Curriculum Framework |
| McCoy et al. (2020) [50], Canada | Commentary | “[…] advocate for a dual-focused approach: combining robust data science-focused additions to baseline health research curricula and extracurricular programs to cultivate leadership in this space.” | Multiple (UME; general) | Educational Program |
| Nagy et al. (2022) [51], USA | Commentary | “[…] argue that foundational ML principles should be taught broadly to medical students across the country.” | Multiple (UME; N/A, CME; N/A) | Educational Program |
| Nguyen & Shetty (2018) [52], USA | Opinion | “[…] [describes the] gap in training [and] where does a radiologist in training begin […]” | PGME; radiology | Educational Program |
| Paranjape et al. (2019) [12], USA | Opinion | “[…] addressed the state of medical education at present and have recommended a framework on how to evolve the medical education curriculum to include AI.” | Multiple (UME, PGME, CME) | Educational Program |
| Park et al. (2019) [53], Korea | Review | “[…] provide a succinct summary of the current state of AI from a medical viewpoint and suggest what medical students should do to prepare for the era of AI in medicine.” | UME; general | Educational Program |
| Sapci & Sapci (2020) [54], USA | Review | “[…] evaluate the current state of AI training and the use of AI tools to enhance the learning experience.” | Multiple (UME, PGME, CME) | Educational Program |
| Tschirhart et al. (2022) [55], Canada | Perspective | “[…] describe an example of a rigorous labelling program using lung ultrasound (LUS) images that confers both AI fluency and domain expertise for interested medical students.” | UME; general | Educational Program |
| Valikodath et al. (2021) [57], USA | Perspective | “[…] explore AI in ophthalmology, perceptions of AI among the medical community, the need to adopt AI in medical education while preserving the humanization of medicine, and recommendations for an AI curriculum for medical students, residents, and fellows in ophthalmology.” | PGME; ophthalmology | Curriculum Framework |
