# Supplementary figures and images for: Curriculum Frameworks and Educational Programs in AI for Medical Students, Residents, and Practicing Physicians: Scoping Review
Source: JMIR Med Educ. 2024 Jul 18;10:e54793. doi: 10.2196/54793 (PMC11294785; doi:10.2196/54793)

### Multimedia Appendix 3. Countries and years of publications included in the review.

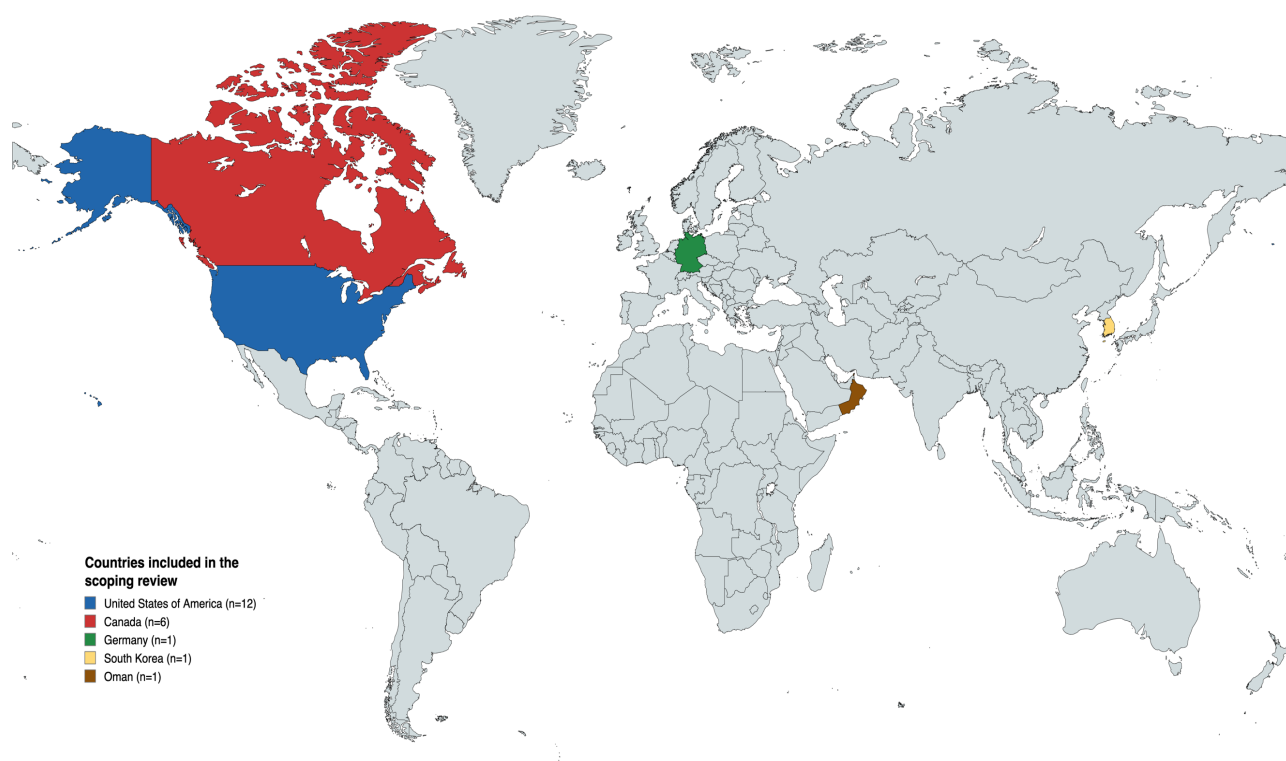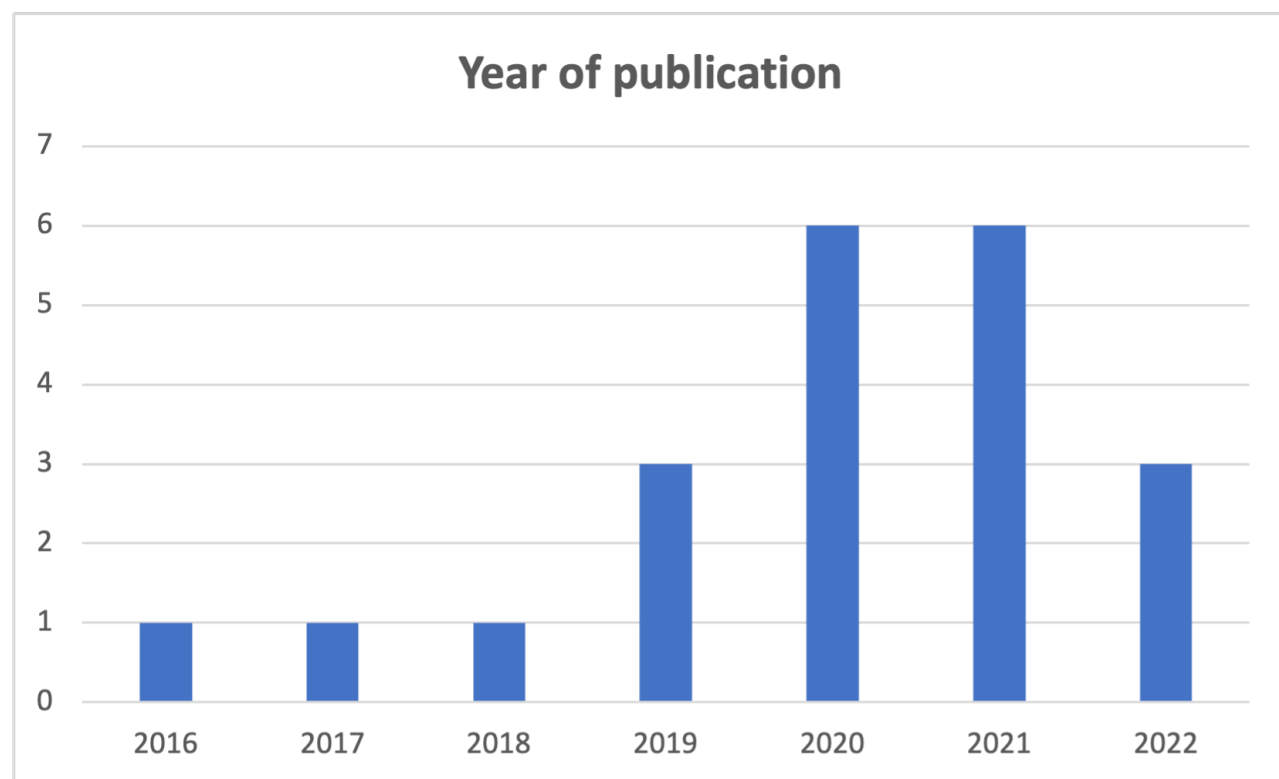

Supplement: Multimedia Appendix 3 [file mededu_v10i1e54793_app3.pdf]
